# EFFECTIVE SOLUTIONS FOR CAREGIVERS OF OLDER ADULTS: A RAPID UMBRELLA REVIEW

**DOI:** 10.1093/geroni/igad104.3100

**Published:** 2023-12-21

**Authors:** Molly McHugh, Ellen Munsterman, HanNah Cho, Mary Naylor

**Affiliations:** The University of Pennsylvania, Philadelphia, Pennsylvania, United States; The University of Pennsylvania, Philadelphia, Pennsylvania, United States; The University of Pennsylvania, Philadelphia, Pennsylvania, United States; The University of Pennsylvania, Philadelphia, Pennsylvania, United States

## Abstract

The negative health impacts confronting over 35 million informal caregivers of older adults in the U.S. are well documented. Although targeted interventions aimed at informal caregivers have demonstrated efficacy in mitigating poor caregiver outcomes, gaps in the literature regarding cost, scalability, and the impact of policy remain. Such knowledge is essential, given the projected growth in the need for caregiving associated with a rapidly aging population coupled with the shrinking of the caregiving workforce. This rapid umbrella review aims to synthesize findings from systematic reviews of randomized clinical trials of interventions that targeted caregivers and specified caregiver outcomes. The National Institute on Minority Health and Health Disparities research framework guided this review to identify gaps in existing intervention research. Databases from diverse disciplinary perspectives, including PubMed, Scopus, APA PsycINFO, ABIM, ATLA, Sociological Abstracts, PAIS, and EconLit, were searched from 2018 to 2023. A total of 52 systematic reviews that met inclusion criteria and were published in English were included in the final review. Reviews described interventions targeting caregivers of older adults with the following conditions: dementia (30), stroke (4), cancer (4), heart disease (1), renal disease (1), mental illness (1), and mixed-diagnoses (11). Major findings reveal varied effects on caregiver burden, quality of life, and mental health, and the growing integration of telehealth interventions. Studies were commonly limited by heterogeneity of interventions and outcomes and small effect sizes. Future intervention development should consider targeting the specific needs of diverse caregivers and scaling effective interventions to broader caregiver populations.

